# Behavioral and Molecular Effects of Thapsigargin-Induced Brain ER- Stress: Encompassing Inflammation, MAPK, and Insulin Signaling Pathway

**DOI:** 10.3390/life12091374

**Published:** 2022-09-02

**Authors:** Sahar Askari, Pegah Javadpour, Fatemeh Sadat Rashidi, Leila Dargahi, Khosrow Kashfi, Rasoul Ghasemi

**Affiliations:** 1 Department of Physiology, Faculty of Medicine, Shahid Beheshti University of Medical Sciences, Tehran 11151-19857, Iran; 2 Neuroscience Research Center, Shahid Beheshti University of Medical Sciences, Tehran 11151-19857, Iran; 3 Neurobiology Research Center, Shahid Beheshti University of Medical Sciences, Tehran 11151-19857, Iran; 4 Department of Molecular, Cellular & Biomedical Sciences, City University of New York School of Medicine, New York, NY 10031, USA; 5 Neurophysiology Research Center, Shahid Beheshti University of Medical Sciences, Tehran 11151-19857, Iran

**Keywords:** Alzheimer’s disease, endoplasmic reticulum stress, apoptosis, inflammation, MAPK, insulin resistance, spatial memory impairment

## Abstract

Accumulation of misfolded proteins, known as endoplasmic reticulum (ER) stress, is known to participate in Alzheimer’s disease (AD). AD is also correlated with impaired central insulin signaling. However, few studies have probed the relationship between memory, central ER stress, inflammation, hippocampal mitogen-activated protein kinase (MAPK) activity and insulin resistance. The present study aimed to investigate the causative role and underlying mechanisms of brain ER stress in memory impairment and develop a reliable animal model for ER-mediated memory loss. Thapsigargin (TG), a known ER stress activator, was centrally administered. The cognitive function of animals was evaluated by the Morris Water Maze (MWM). To verify the induction of central ER stress, we investigated the mRNA expression of UPR markers in the hippocampus. In addition, the activation of ER stress markers, including Bip, CHOP, and some related apoptosis and pro-inflammatory proteins, such as caspase-3, Bax, Bcl-2, TNF-α, MAPK, and insulin signaling markers, were assessed by Western-blots. The results demonstrated that TG impairs spatial cognition and hippocampal insulin signaling. Meanwhile, molecular results showed a concurrent increment of hippocampal UPR markers, apoptosis, P38 activity, and TNF-α. This study introduced TG-induced ER stress as a pharmacological model for memory impairment in rats and revealed some underlying mechanisms.

## 1. Introduction

Today, Alzheimer’s disease (AD), a leading cause of dementia, is a prevalent and aged-related progressive neurodegenerative disorder affecting nearly 50 million people worldwide. It is estimated that this number will be over 152 million by 2050 [1]. Two pathological hallmarks of AD are an aggregation of beta-amyloid (Aβ) peptides and the formation of neurofibrillary tangles (NFTs) from tau protein [2,3]. Indeed, tau protein normally causes microtubule stabilization to vesicle trafficking and axonal elongation [4]. In diseased brains, tau proteins are first hyper-phosphorylated and then solubilized and polymerized to form NFTs [5,6]. Various pharmacological and genetic models have been used to study AD’s pathophysiology and introduce potential therapeutic targets. Some of these models, such as amyloid-beta (Aβ) administration [7,8,9,10,11,12,13], amyloid precursor protein (APP), and/or PS1-overexpressing transgenic models [14], simulate the pathology of AD through Aβ accumulation. In other experimental models of AD, memory deficit is modeled by the induction of neuroinflammation, glucose metabolism alteration, or cholinergic dysfunction by the administration of lipopolysaccharide (LPS) [15], STZ [16], scopolamine [17,18,19], respectively. Each of these models relies on one of the current hypotheses on AD etiology and provides some pathological characteristics of AD. Endoplasmic reticulum (ER) impairment is one of the important pathological characteristics reported in some of the AD experimental models [20,21,22,23,24,25,26,27], and is even considered a pathological feature in the human AD brain [28,29,30,31]. ER is a cellular site crucial for the synthesis, folding, and maturation of numerous transmembrane and secreted proteins [32]. In response to the accumulation of misfolded proteins in the ER, a series of signaling pathways is triggered to maintain protein folding homeostasis, known as the unfolded protein response (UPR) [33]. Inositol-requiring protein-1 alpha (IRE1α), PKR-like ER kinase (PERK), and the activating transcription factor-6 (ATF6) are three sensors controlling these pathways [33,34]. Activating transcription factor-4 (ATF4) is activated by PERK and affects the transcription of specific UPR target genes, including the C/EBP homologous protein (CHOP) [35,36], which leads to apoptosis, activation of pro-apoptotic factors, and inhibition of anti-apoptotic factors [37]. Activation of IRE1α precedes the conversion of an un-spliced X-box binding protein 1 (us-XBP1) protein to a spliced XBP1 (s-XBP1) protein [38]; thus, an elevated s-XPB1/us-XBP1 mRNA ratio indicates IRE1α pathway activation [39,40]. Thapsigargin (TG) is extracted from a plant, *Thapsia garganica,* [41], and is a non-competitive inhibitor of the sarco/endoplasmic reticulum Ca^2+^ ATPase (SERCA) [42]. Thus, it can deplete ER calcium stores, causing ER stress [43]. Using ER stress inhibitors could alleviate symptoms of ER stress. For example, 4-phenylbutyric acid (PBA) is one of the best ER stress blockers. It is classified as a chemical chaperon by the US Food and Drug Administration (FDA) and is an approved drug for treating urea cycle disorders [44]. PBA could relieve ER stress by improving ER capacity, preventing misfolded protein accumulation, and decreasing the mis-trafficking of mutant proteins [45]. PBA also suppresses caspase-3 activation [46,47].

Given the fact that ER stress is a molecular feature of AD, few studies have used ER stress induction to model memory impairment [48,49,50,51], and even less is known about the molecular mechanisms underlying the effects of ER stress on cognitive function.

To find the potential molecular mechanisms, we considered a growing body of studies suggesting the contribution of apoptosis [52], neuroinflammation [53], and brain insulin resistance [54,55] in AD. These phenomena (apoptosis [56], neuroinflammation [57], and brain insulin resistance [58,59,60] are involved in ER stress too. Different signaling pathways, such as mitogen-activated protein kinase (MAPK) network pathways, participate in each of these phenomena [61,62,63,64,65]. MAPK signaling pathways have also been involved in the pathology of AD [66,67,68] and ER stress [69,70]. Moreover, previous evidence suggests the involvement of c-Jun N-terminal kinase (JNK) and P38 MAPK cascades in ER stress-induced apoptosis [71]. By JNK activation, ER stress-mediated alterations can dysregulate the brain insulin signaling pathway [72] affecting learning and memory [73].

Therefore, in the current study, we aimed to investigate the effects of Thapsigargin-induced brain ER stress on spatial learning and memory and explored the potential molecular mechanisms through which central ER stress results in memory deficit.

## 2. Results

### 2.1. Behavioral Results

**Figure 1:**
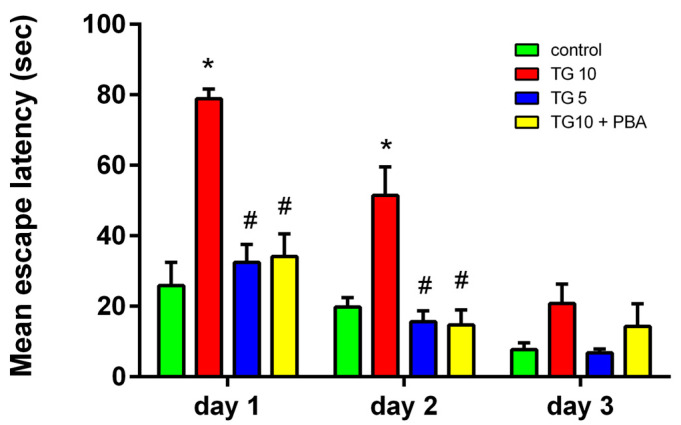
Effects of TG (5, 10 µg) and TG10 + PBA on the escape latency to arrive at a hidden platform in a water maze as a function of the training day. These data reveal that the escape latency is longer in TG10-treated animals during days 1 and 2 of training. * *p* < 0.001 versus the control group. # *p* < 0.001 versus the TG10 group. Data are represented as mean ± SEM (N = 7–9). Thapsigargin (TG), 4-Phenylbutyric acid (PBA).

The animals were trained for three consecutive days to determine how central ER stress affected their spatial learning and memory. Although all experimental groups showed a decrease in the time needed to find the platform (Figure 1), two-way ANOVA indicated a significant difference between groups in escape latency to the platform (F (3, 28) = 21.78, *p* < 0.0001). In the next step, post-hoc comparisons by Tukey’s test revealed a significant increase in escape latency to the platform between the TG10-treated and the other three groups on the first and second days of training for the MWM. There was no significant change between the TG5 and TG10 + PBA groups compared to the control group in the escape latency to the platform. However, both groups showed a remarkable reduction versus the TG10 group (Figure 1).

**Figure 2:**
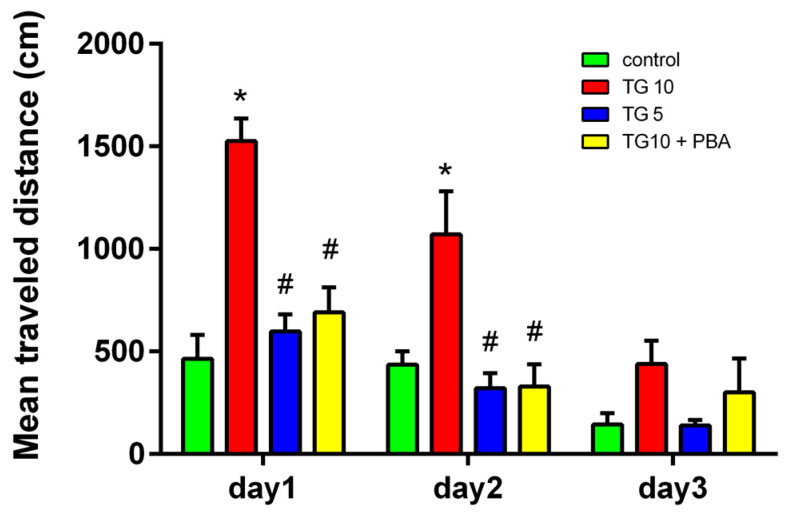
Effects of TG (5, 10 µg) and TG10 + PBA on distance traveled in the water maze training trials. In the TG10-treated group, the traveled distance is greater during days 1 and 2 of training. * *p* < 0.001 versus the control group. # *p* < 0.001 versus the TG10 group. Data are represented as mean ± SEM (N = 7–9). Thapsigargin (TG), 4-Phenylbutyric acid (PBA).

Similarly, a two-way ANOVA analysis of the total distance that animals swam to reach the platform revealed a significant difference between groups (F (3, 28) = 17.46, *p* < 0.0001). In addition, the Tukey’s post-hoc test indicated that on day1 and 2, animals in the TG10 group swam a greater distance to reach the platform; however, no significant difference was evident between the TG5, TG10 + PBA, and control groups. However, the TG5 and TG10 + PBA groups showed a significant decline compared to the TG10 group (Figure 2).

**Figure 3:**
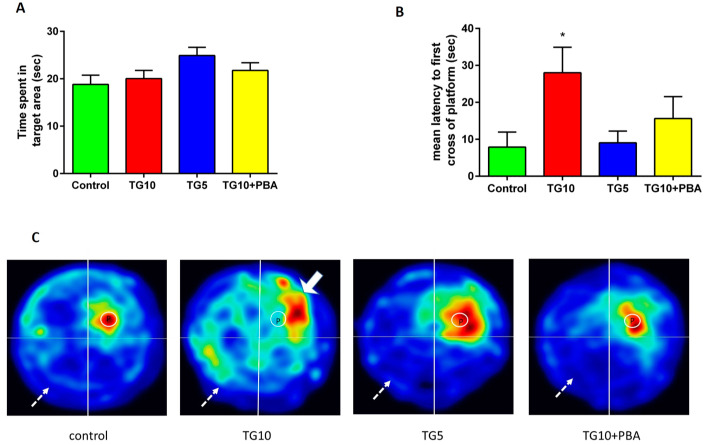
The effects of TG (5, 10 µg) and TG10 + PBA on (**A**) the time spent in the target quadrant, (**B**) the latency to the first cross of the platform, and (**C**) the mean heat-map visualization of animal performance in the probe test. The solid arrow is showing a hot zone in target zone showing animal spend more time in this area and dashed lines is showing the wrong zone which is less explored. * Significantly different from the control (*p* < 0.05). Data are represented as mean ± SEM (N = 7–9). Thapsigargin (TG), 4-Phenylbutyric acid (PBA).

To evaluate memory retrieval, rats were assessed using a probe test. One-way analysis of the time spent in the target quadrant failed to show a significant difference between the groups (F (3, 28) = 2.397, *p* = 0.0892) (Figure 3A). Another criterion extracted in the probe test was the latency to reach the platform for the first time. Here, one-way ANOVA showed a significant difference (F (3, 28) = 3.350, *p* < 0.05) between the groups (Figure 3B). In the next step, post-hoc comparisons by Tukey’s test revealed a significant (*p* < 0.05) difference between TG10-treated rats and the control group, but this was not the case for the control and TG5 and TG10 + PBA groups.

Figure 3C shows each group’s mean heat-map visualization of animal performance in the probe test. In this figure, the platform’s location is depicted by a circle. As it is evident, the TG10 rats were less aware of the platform location. In other words, despite the nearly similar time spent in the target quadrant, the TG10-treated rats mainly swam in the periphery of the maze (as shown by the solid arrow). In contrast, the other groups primarily targeted the location of the platform. This figure explains why the statistical analysis of time spent in the target quadrant was not significant, while the other criterion (first latency to platform) indicated a significant difference. In addition, this figure demonstrates that animals with cognitive retardation spent more time in the opposite quadrant. At the same time, the other groups explored this quadrant less frequently (as shown with the lighter color and dashed arrows).

**Figure 4:**
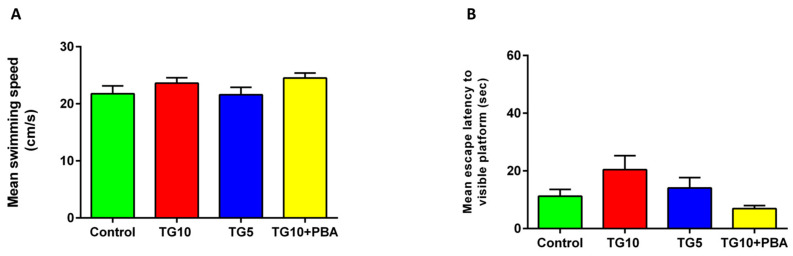
Effects of TG (5, 10 µg) and TG10 + PBA on the mean of (**A**) the animals’ swimming speeds and (**B**) the animals’ abilities to locate the visible platform. There was no observable difference between the groups. Data are represented as mean ± SEM (N = 7–9). Thapsigargin (TG), 4-Phenylbutyric acid (PBA).

Additionally, to see if central ER stress induction affects the rat’s motor function, the mean swimming speed of the animals was calculated in the probe test. Results are shown in Figure 4A. Again, one-way ANOVA revealed no difference between the groups (F (3, 28) = 1.438, *p* = 0.2528).

Moreover, the visible platform test was carried out 4 days after the probe test to assess the animal’s health and performance of sensory-motor, visual system, and motivation. These results were also analyzed by one-way ANOVA, and no significant difference was observed between the groups (F (3, 28) = 2.613, *p* = 0.0709) (Figure 4B).

### 2.2. Results of PCR Assessments

**Figure 5:**
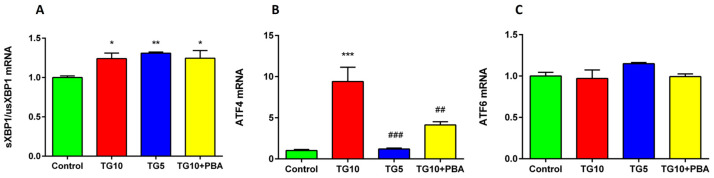
Effects of TG (5 & 10 µg) and TG10 + PBA on (**A**) sXBP1/usXBP1 mRNA expression, (**B**) ATF4 mRNA expression, and (**C**) ATF6 mRNA expression. * *p* < 0.05, ** *p* < 0.01, and *** *p* < 0.001 versus the control group. ## *p* < 0.01 and ### *p* < 0.001 versus the TG10 group. Data are represented as mean ± SEM (N = 3–4). Thapsigargin (TG), 4-Phenylbutyric acid (PBA).

To assess ER stress induction, we measured the mRNA expression for mRNA levels of s-XBP1, us-XBP1, ATF4, and ATF6. S-XBP1/us-XBP1 ratio: A significant difference between the groups was identified using one-way ANOVA (F (3, 10) = 7.751, *p* = 0.0058). Tukey’s post-hoc test showed that this ratio was elevated in the hippocampus of the TG10, TG5, and TG10 + PBA groups versus the control (Figure 5A).

ATF4: The result of the ANOVA and Tukey’s post-hoc test indicated that the TG10 group was significantly different from the other groups (F (3, 10) = 26.41, *p* = 0.0486). This important ER stress-induced apoptotic pathway was only activated in the TG10 group. However, the TG5 and TG10 + PBA groups showed a meaningful decline in ATF4 mRNA. There was no difference between the TG5, TG10 + PBA, and control groups (Figure 5B).

ATF6: Statistical analysis indicated no difference among the groups (F (3, 10) = 0.6112, *p* = 0.6230) (Figure 5C).

### 2.3. Results of Western Blot Analysis

Western blots were performed to investigate the impact of central TG administration on molecular alterations of protein expression of hippocampal CHOP, Bip, caspase-3, Bax, and Bcl2.

**Figure 6:**
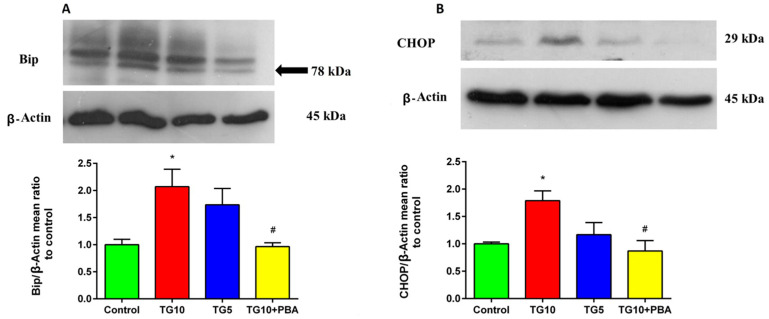
Effects of TG (5, 10 µg) and TG10 + PBA on (**A**) Bip and (**B**) CHOP. * *p* < 0.05 versus the control group. # *p* < 0.05 versus the TG10 group. Data are represented as mean ± SEM (N = 4). For clarity, the background to panel A was made lighter in Photoshop; full uncropped blots are provided as Supplementary Material. Thapsigargin (TG), 4-Phenylbutyric acid (PBA).

For Bip and CHOP, we evaluated the activity of Bip protein as an important marker for ER stress. The results of the ANOVA and Tukey’s post-hoc test revealed that the TG10 group had a significantly higher expression of Bip from that of the control and TG10 + PBA groups (F (3, 12) = 5.777, *p* = 0.0111) (Figure 6A). In addition to Bip, the activity of the CHOP protein, another important marker for ER stress, was also assessed. Statistical analysis showed a significant difference among the groups (F (3, 12) = 5.590, *p* = 0.0124). Furthermore, according to post-hoc results, the level of CHOP protein in the TG10 group showed a significant increase compared to the control and TG10 + PBA groups (Figure 6B). Collectively, no significant changes were observed between TG5, TG10 + PBA, and control groups in these ER stress biomarkers. However, PBA significantly reduced them compared to the TG10 group. There was no significant difference between the TG5 group and the TG10 and TG10 + PBA group (Figure 6).

**Figure 7:**
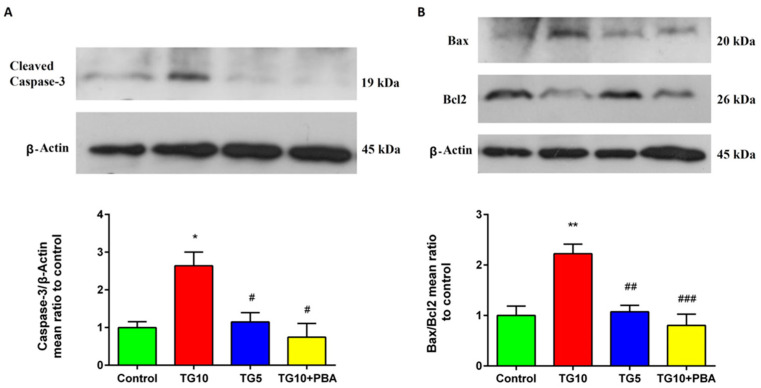
Effects of TG (5, 10 µg) and TG10 + PBA on (**A**) the caspase-3 and (**B**) Bax/Bcl2. * *p* < 0.05 and ** *p* < 0.01 versus the control group. # *p* < 0.05, ## *p* < 0.01 and ### *p* < 0.001 versus the TG10 group. Data are represented as mean ± SEM (N = 4). The representative blots for Figure 7 and Figure 9A were obtained from the same gel, therefore the β-Actin blot represented in these figures is the same. Thapsigargin (TG), 4-Phenylbutyric acid (PBA).

To investigate if apoptotic pathways are induced by central ER stress, we evaluated expressions of caspase-3 and Bax/Bcl2. While the activity of caspase-3 was increased in the TG10 group compared to the TG5, TG10 + PBA, and control groups (F (3, 10) = 8.679, *p* = 0.0039), there was not a remarkable difference between the others. This means that TG5 does not affect the activity of caspase-3 and that PBA mediated reduction of hippocampal content of caspase-3 through ER stress amelioration in TG10 rats (Figure 7A). To determine the effect of centrally induced ER stress on the mitochondrial apoptotic process, we measured the expression of Bax, a pro-apoptotic protein, and Bcl2, an anti-apoptotic protein, and calculated the Bax/Bcl2 ratio. A one-way ANOVA followed by Tukey’s post-hoc test indicated that this ratio was meaningfully higher in the TG10 treated group compared with the control, TG5, and TG10 + PBA groups (F (3, 12) = 12.09, *p* = 0.0006). As well as active caspase-3, the Bax/Bcl2 ratio did not show any meaningful changes in the TG5 and TG10 + PBA groups compared to the control group. However, they both showed a remarkable Bax/Bcl2 ratio reduction compared to the TG10 group (Figure 7B).

**Figure 8:**
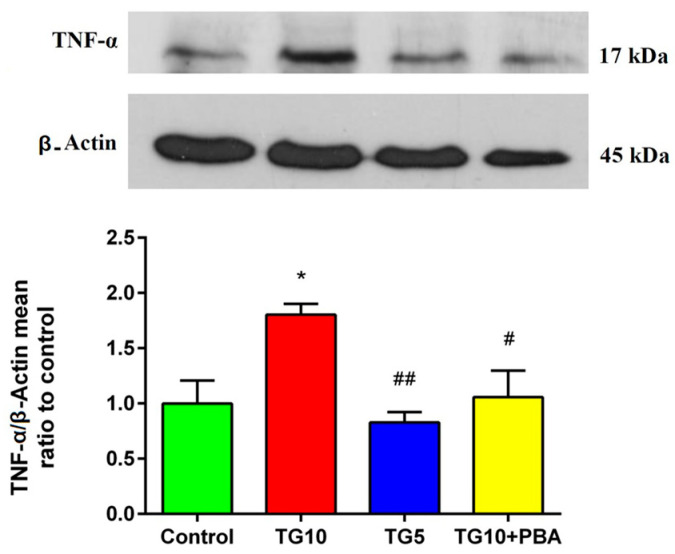
Effects of TG (5, 10 µg) and TG10 + PBA administration on TNFα. * *p* < 0.05 versus the control group. # *p* < 0.05 and ## *p* < 0.01 versus the TG10 group. Data are presented as mean ± SEM (N = 4). Thapsigargin (TG), 4-Phenylbutyric acid (PBA).

To explore the relationship between inflammation and central, induced ER stress, we assessed TNF-α protein expression. One-way ANOVA showed a significant difference in TNF-α between the groups (F (3, 12) = 6.239, *p* = 0.0085). In addition, Tukey’s post-hoc test showed that the content of hippocampal TNF-α increased in the TG10 group versus the others. There was no remarkable difference between the TG5, TG10 + PBA, and control groups. However, the TG5 and TG10 + PBA groups displaced a meaningful reduction in TNF-α versus the TG10 group (Figure 8).

**Figure 9:**
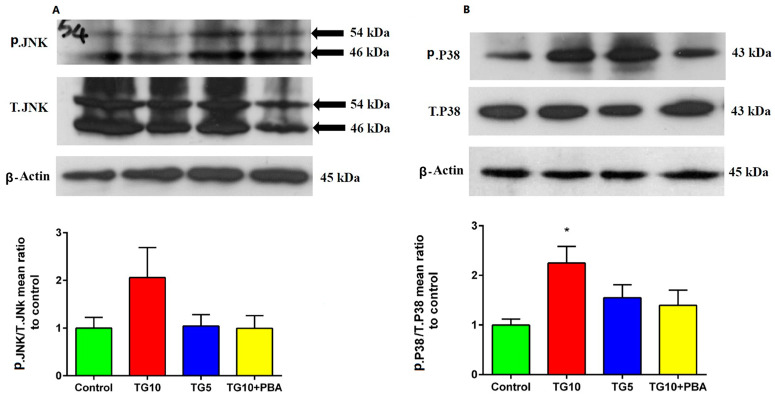
Effects of TG (5, 10 µg) and TG10 + PBA on (**A**) JNK and (**B**) P38. * *p* < 0.05 versus the control group. Data are presented as mean ± SEM (N = 4). Please note that since representative blots for Figure 7 and Figure 9A were obtained from the same gel, the β-Actin blot represented in these figures are the same. For clarity, the background to panel A was made lighter in Photoshop. Full uncropped blots are provided as Supplementary Material. Thapsigargin (TG), 4-Phenylbutyric acid (PBA).

To explore whether MAPK and insulin signaling pathway alterations participate in the abovementioned effects of central ER stress, we considered JNK, P38, and IRS1ser307 in the hippocampal lysate. Statistical analysis failed to show a meaningful difference among the groups (F (3, 28) = 1.910, *p* = 0.1818), but an incremental trend was seen in the TG10 group (Figure 9A). Regarding the P38 phosphorylation state, statistical analysis indicated a significant difference between the groups (F (3, 12) = 3.746, *p* = 0.0415). In addition, a Tukey’s post-hoc test showed that the content of hippocampal p.P38 in the TG10 group was enhanced compared to the control group (*p* < 0.05). However, there was no significant difference between other groups in P38 phosphorylation (Figure 9B).

**Figure 10:**
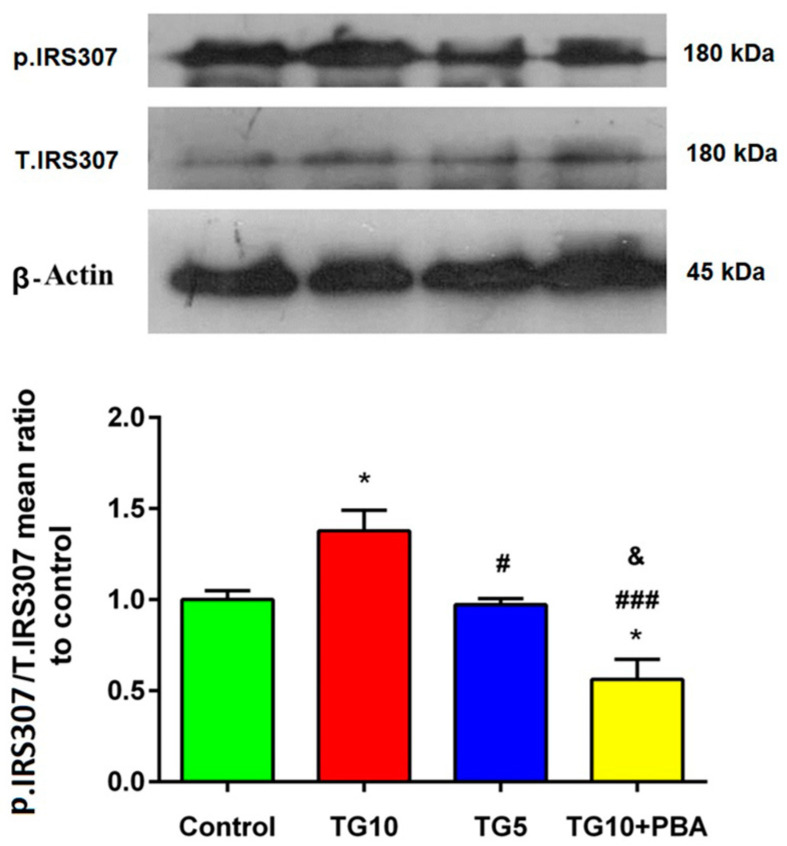
Western blot analysis revealing the impacts of TG dosages (10, 5) administration on IRS1307. * *p* < 0.05 versus the control group. # *p* < 0.05 and ### *p* < 0.001 versus the TG10 group. & *p* < 0.05 versus TG5. Data are presented as mean ± SEM (N = 4). Thapsigargin (TG), 4-Phenylbutyric acid (PBA).

A one-way ANOVA followed by Tukey’s post-hoc test showed that the hippocampal content of Insulin receptor substrate 1 (IRS1ser307) increased in the TG10 group versus the others (F (2, 9) = 9.254, *p* = 0.0066). There were no main changes between the TG5 and control groups. The TG10 + PBA group showed a significant reduction in IRS1 phosphorylation compared to the control, TG10, and TG5 groups (Figure 10).

## 3. Discussion

In the present work, we provide solid evidence showing that TG-mediated central ER stress causes the deterioration of spatial learning and memory. Interestingly, the present study also showed that co-treatment of TG and PBA, an inhibitor of TG-induced ER stress [74,75], could nullify these effects, proving that the cognitive dysfunction is triggered by ER stress, and is not a non-specific effect of TG. Several experimental studies have suggested a potential relationship between ER stress and AD [76,77,78,79,80]. Nevertheless, fewer experimental models of AD, induced by ER stress, are available for direct study of this relationship [48,49,50,51]. In line with our findings, Lourenco and colleagues have investigated the effect of TG on non-spatial recognition memory using Novel Object Recognition (NOR) tasks in mice [48]. Moreover, other studies have reported that central induction of ER stress with Tunicamycin (TM), another recognized ER stress inducer, impaired spatial memory [49,50,51]. In addition to the differences in the type of ER stress inducer and its mechanism of action, studies on TM have shown discrepancies in memory tests and different memory phases. For example, in Wang et al.’s study, cognitive function was assessed by the Y maze test, and they reported that a high dose of TM causes memory dysfunction [50]. Meanwhile, Lin et al. depicted that TM impaired the retrieval phase in spatial memory. However, this study did not assess memory acquisition or consolidation phases [51].

To further verify that the loss of memory is a consequence of ER stress, we measured the expression of s-XBP1, us-XBP1, ATF4, and ATF6 genes, and also determined the protein levels of Bip and CHOP as important ER stress markers [81,82,83,84], in the hippocampus. Furthermore, to investigate the involved mechanisms, we assessed apoptosis and inflammation by assessing Caspase-3, Bax, Bcl2, and TNF-α. Our results showed that central injection of TG10 induces hippocampal ER stress in rats, characterized by an increase in the sXPB1/usXBP1 mRNA ratio, ATF4 mRNA, CHOP, and Bip expression when compared to control rats. Bip, a well-known marker for ER stress, is at a lower level in ER under normal conditions [85]. Bip dissociation from transmembrane ER stress sensors (IRE1α, PERK, and ATF6) is a crucial step in their activation. There is evidence that activation of ATF6 and s-XBP1 can upregulate Bip mRNA [39,86]. In the present study, we demonstrated that the expression level of the Bip protein and s-XBP1/u-XBP1 mRNA increased in the TG10 group, but the expression of ATF6 mRNA did not show significant elevation. This means upregulation of Bip can result from s-XBP1 enhancement, which is in line with previous reports showing that the expression of Bip was highly related to the amount of XBP1 mRNA [86]. In line with our study, a recent report showed that memory impairment could be linked to Bip elevation in the hippocampus [87]. Furthermore, previous reports have demonstrated that Bip protein is enhanced in the hippocampus of AD patients [21,30,88] and animals that were treated with ER stress inducers [89,90]. These findings are in agreement with our results, which indicated that the deterioration of spatial learning and memory was accompanied by a significant increase in the expression of the Bip protein. In addition, the present results showed an elevated hippocampal ATF4 mRNA expression. In response to ER stress, ATF4 mediates CHOP gene enhancement, which, in turn leads, to activation of pro-apoptotic proteins such as BAX and Caspase-3 [91], reduction of BCl2 as an anti-apoptotic protein [92], and induction of several apoptotic pathways [35]. Furthermore, mounting evidence has shown that elevated CHOP expression is associated with memory loss. Accordingly, Zhang and colleagues published that induction of ER stress by hyperglycemia raised the level of CHOP protein in the hippocampus and led to spatial learning dysfunction [80]. Moreover, in another study, Wang et al. observed that the hippocampal CHOP level was enhanced in severe ER stress induced by TM, and this elevation coincided with cognitive impairment [50]. In line with these studies, our outcomes also suggest that, at least in part, the promotion of ATF4 might participate in neuronal death and memory impairment in the TG10 group by CHOP activation.

It is well-established that cognitive dysfunction reflects neuronal death in the hippocampus [93], a phenomenon demonstrated specifically in AD patients [94]. Thus, it is conceivable that ER stress inducers (specific or nonspecific) may lead to cognitive impairment through hippocampal neuronal apoptosis [80,95]. It is established that caspase-3 activity is essential for apoptosis, and that cleaved caspase-3 may be considered a general marker for apoptosis [96]. Caspase-3 has a remarkable role in cognitive impairment in AD patients by increasing synaptic degeneration and synaptic loss [97]. Studies have demonstrated that tunicamycin, an ER stress inducer, could also elevate caspase-3 activity, which is consistent with apoptotic cell death [98]. Our Western blots showed increases in cleaved-caspase-3 expression in the TG10 group only. In line with our findings, Hongna et al. reported that ER stress induction by arsenite enhanced apoptosis through caspase-3 expression, which was accompanied by neuronal death in the hippocampus, a decrease in learning ability, and memory deficit acceleration [99]. Like caspase-3, Bax and Bcl2 play a pivotal role in regulating neuronal apoptosis, and the ratio between anti-apoptotic and pro-apoptotic factors determines the fate of the neurons [100]. Any agent with an enhancement effect on Bax/Bcl2 may lead to apoptosis [101]. Bax is another protein that contributes to ER stress-mediated apoptosis [102]. ER stress stimulates Ca2+ depletion from ER stores and accumulation of Ca2+ in mitochondria, leading to pro-apoptotic mitochondrial alterations such as re-localization of Bax to mitochondria [103]. Moreover, another apoptosis mechanism during ER stress conditions is related to the direct inhibition of Bcl2 by activated CHOP [104,105]. CHOP also promotes Bax translocation from the cytosol to the mitochondria [104]. Furthermore, neuronal apoptosis and increased Bax/Bcl2 in the hippocampus are consistent with cognitive impairment [106,107,108]. Again, a recent study found that inhibiting ER stress-mediated apoptosis markers (including CHOP, active caspase-3, Bax/Bcl2, and JNK) improves spatial learning ability [109]. In the present study, Bax/Bcl2 ratio was increased in the TG10 group, which means this ratio inclined toward Bax elevation. Based on our studies, memory dysfunction in our experiment can be related to central ER stress-mediated apoptosis.

ER stress also stimulates TNF-α secretion [110], and can even induce neuronal inflammation [111]. Wang et al., using cultured hippocampus neurons, showed that increased TNF-α accompanies ER stress induction. However, PBA can attenuate ER stress-induced inflammation [111]. Studies have showed that during ER stress, ATF6 and IRE1α play an important role in inflammation. ATF6 can activate NF-κB, which in turn induces pro-inflammatory factors such as TNF-α [112]. TNF-α is also encoded by XBP1 mRNA [113]. In the current study, although the expression of ATF6 mRNA did not show a significant effect in the TG10 group versus the other groups, TNF-α was increased. Therefore, we propose that XBP1 splicing can be enough to promote TNF-α production. Furthermore, studies have reported that elevated TNF-α can lead to memory impairment [48] and cognitive deficit [53], which contributes to different brain disorders, including AD, trauma, and Parkinson’s disease [54]. In line with these studies, it is possible that elevated TNF-α is one of the involved factors in memory impairment in the TG10 group. On the other hand, hyper activation of MAPKs (JNK, P38, and ERK) through ER stress can also mediate inflammation [114,115]. PERK and ATF6 induce ERK and P38, while all three sub-pathways of UPR are involved in JNK induction [113]. In addition, JNK and P38 MAPK cascades play an important role in ER stress-induced apoptosis [71]. Inhibition of these kinases leads to attenuation of contributing proteins to apoptosis, e.g., CHOP and cleaved-caspase-3 [70]. P38 and JNK are important modulators of events closely related to memory impairment, including apoptosis, tau phosphorylation, and Aβ generation [116]. A recent study also showed that suppression of JNK, one of the main factors involved in ER stress-induced apoptosis in the parietal cortex and hippocampus could be effective in learning and memory improvement [109]. Our current findings consistently demonstrated that the activity of P38 increased in the TG10 animals versus the control group. Despite the elevated trend of JNK activity, we did not find a statistically significant change in the TG10 group compared to the others. Thereby, it is possible to propose that enhanced phosphorylation of P38 and JNK could be other important factors in ER stress-mediated memory impairment. Moreover, evidence supports that these two stress kinases phosphorylate IRS on serine 307, disrupting the insulin signaling pathway [58,99]. IRS1, as an insulin receptor substrate, has various tyrosine and serine phosphorylation sites. Therefore, an increase in phosphorylation of IRS1 on serine residues leads to insulin resistance and a decrease in Akt (a downstream molecule in the insulin signaling pathway) phosphorylation [117,118].

In the present study, we provide evidence that phosphorylation of IRS ser307 in the TG10 group was enhanced, which sheds light on the impaired insulin signaling. In peripheral insulin resistance, TNF-α and JNK signaling activation is an important mechanism [119]. Previous studies have established that ER stress can develop peripheral and brain insulin signaling impairment through JNK activation [72,120,121]. On the other hand, because of the acute effects of insulin on synapse formation and maintenance [122], it is conceivable that brain insulin resistance impairs learning and memory by diminishing hippocampal plasticity and neurodegeneration [73]. By these effects, it is well documented that brain insulin resistance plays a crucial role in AD [122]. So, it is possible to consider the involvement of the central brain insulin signaling pathway in ER stress-induced memory deficit.

Finally, it is essential to note that in almost all of our molecular findings (except the s-XBP1/us-XBP1 mRNA ratio), PBA treatment neutralized all effects of TG on TG10 rats. However, similar to the PBA results, enhancement of the s-XBP1/us-XBP1 mRNA ratio was seen in the TG5 group. As previously mentioned, enhancement of the s-XBP1/us-XBP1 ratio implies the activation of the IRE1α pathway [39,40]. Generally, activation of IRE1α is associated with pro-survival pathways to adapt to ER stress, whereas the PERK/ATF4/CHOP pathway is related to cell death [123]. Therefore, the elevated s-XBP1/us-XBP1 ratio mRNA in only the TG5 and PBA-treated groups may indicate the involvement of survival responses to restore ER capacity. Moreover, it is important to note that the effects of TG could be dose-dependent [98]. Thus, it is possible that TG at the dose of 5 µg was not potent enough for ER stress induction.

## 4. Materials and Methods

### 4.1. Animals

Thirty-two adults male Wistar rats weighing 280–320 g were used in the study. Animals were obtained from the animal house of the Neuroscience Research Center, Shahid Beheshti University of Medical Science. The animals were caged with free access to food and water under standard laboratory conditions with 12 h light-dark cycle (light from 7 a.m. to 7 p.m.) and controlled room temperature (25 ± 2 °C). The rats were randomly divided into four groups (n = 7–9 for behavioral test, n = 3–4 for PCR test, n = 4 for Western blot tests). The Control group received 4 μL Dimethyl sulfoxide (DMSO) as a vehicle through icv injection. The Thapsigargin 5 (TG5) group received a single dose of TG (5 µg) icv. The Thapsigargin 10 (TG10) group received a single dose of TG (10 µg) icv. The 4-Phenylbutyric acid + Thapsigargin 10 (TG10 + PBA) group, which received an intraperitoneal PBA (200 mg/kg) after TG (10 µg). All experiments and methods were conducted according to the standards of the Ethics committee of the Shahid Beheshti University of Medical Sciences (Code: IR.SBMU.MSP.REC.1396.745).

### 4.2. Materials

TG (10522) and PBA (P21005) were purchased from Cayman Chemicals (Ann Arbor, MI, USA) and Sigma-Aldrich (Saint Louis, MO, USA), respectively. Western blot antibodies, including BiP (3183), CHOP (5554), Caspase-3 (9662), Bax (2772), TNFα (3707), p.JNK (4671), T.JNK (9252), p.P38 (9211), T. P38 (8690), p.IRS1-ser307 (2381), T.IRS (2382), β-Actin (4970), and secondary HRP conjugated (7074), were purchased from Cell Signaling Technology (Danvers, MA, USA), and Bcl-2 (ab59348) was obtained from Abcam (Waltham, MA, USA). The Amersham ECL select (RPN2235) reagent kit, Halt™ Protease and Phosphatase Inhibitor Cocktail (78440) and PVDF membrane (IPVH00010) were purchased from GE Health Care and Millipore, respectively. PCR materials were YTzol (Yekta Tajhiz Azma, Tehran, Iran, #YT9065), total RNA isolation kit (RiboEX GeneAll, Seoul, South Korea), 301-902), cDNA Synthesis Kit (Yekta Tajhiz Azma, Tehran, Iran, #YT4500) and SYBR Green Real-Time PCR Master Mix (2×) (Ampliqon, Copenhagen, Denmark).

### 4.3. Drugs Preparation and Administration

#### 4.3.1. TG Microinjection

TG is soluble in organic solvents such as DMSO, ethanol, and acetone. However, it has a very short half-life in solution form. To store TG for longer periods, 1 mg of TG was dissolved in 1 mL of acetone and was then aliquoted (each aliquot contained 20 µg of TG in 20 µL of the solution). In the next step, these aliquots were dried under a stream of nitrogen gas and precipitates were stored at −80 °C. To keep the injection volume constant for TG administration, each aliquot was thawed at room temperature and dissolved in 8 μL of DMSO (2.5 µg/µL), and then 4 μL was administered to each rat through an icv injection route. For the TG5 group, each aliquot was dissolved in 16 μL of DMSO (1.25 µg/µL), and then 4 μL was administrated to each rat as above.

#### 4.3.2. PBA Administration

PBA is soluble in organic solvents, so 200 mg of PBA was freshly dissolved in 100 μL of DMSO + 500 μL of normal saline, and then each rat, in experimental group 4 received 200 mg/kg, intraperitoneally after surgery.

### 4.4. Stereotaxic Surgery

Animals were randomly selected and anesthetized via intraperitoneal injection of mixed Ketamine (100 mg/kg) and Xylazine (10 mg/kg). Based on the Paxinos brain atlas, the left lateral ventricle (AP: −0.84, ML: −1.5 and DV: −3) was located and, after skull penetration, a single dose of the drugs (DMSO or TG in DMSO) was directly injected by Hamilton syringe (10 µL). This microinjection was done at a constant speed of 0.5 µL/min. Thereafter, the animals were given 7 days of recovery before being tested in the Morris Water Maze (MWM) (days 7–10).

### 4.5. Behavioral Test

#### MWM Apparatus

The MWM is a behavioral test used in the present study to evaluate hippocampal-dependent spatial learning and memory. The maze is a black circular pool (height: 70 cm, diameter: 150 cm) divided into four equal quadrates (1, 2, 3, 4 zones). A hidden platform (10 cm diameter, 23 cm height) was located 2 cm beneath the surface of the water in the center of the 2nd zone (target zone). During the test, 2-D and 3-D distinctive and distal visual cues (bookshelves, computer, and posters) were fixed at different locations to aid in the animal’s orientation. A CCD camera was placed above the center of the pool to record the behavior of the swimming animal, and the data were analyzed by a computerized system (Noldus, EthoVision XT 11) (Noldus Information Technology, Wageningen, The Netherlands). Parameters such as escape latency to reach the hidden platform across trials and distance moved to find the platform were extracted during three days of training. Additionally, swim speed, time spent in the target quadrant, and latency of first entrance to the platform location were recorded during the probe test, and escape latency to reach the visible platform was calculated during the visible test.

### 4.6. Procedure

To assess the rats’ spatial learning and memory performance, we designed an MWM test for four days. On the first day before initiation of the trials, rats were placed on the platform for 20 s to get adjusted, and then the learning trials were started. Each of the first three training days consisted of four trials. In each trial, the rats were released into the water at different points and were allowed to find the hidden platform, after which they were permitted a 20-second rest on the platform before the subsequent trial. If the rats could not find the hidden platform in 90 s, they were guided to it by the experimenter.

On the fourth day, the probe (retention test) and visible tests were conducted. In the probe test to assess reference memory. The animals were released into water from the zone located opposite the target zone, but the platform was absent, and they were allowed to swim for 60 s while study parameters were extracted. The visual test was done to evaluate the rat’s motivation, visual ability, and sensorimotor abilities. In this test, the platform was covered by a piece of aluminum foil and then placed in the opposite zone above the water level. The rats were then released into the pool in four trials and allowed to find the visible platform in the same manner as the first three days of training.

### 4.7. Tissue Preparation

For molecular studies, at the end of the MWM test, the animals were anesthetized by CO_2_ inhalation, and their hippocampi were isolated, snap frozen by liquid nitrogen, and then stored at −80 °C.

### 4.8. RNA Isolation and qPCR

Total RNA was isolated from the hippocampus using YTzol according to the manufacturer’s recommendations. The purity and concentration of total RNA were measured by a spectrophotometer NanoDrop 2000 (Thermo Scientific, Wilmington, DE, USA). cDNA synthesis was carried out using 500 ng of total RNA and oligo(dT) primer according to the kit instructions, incubated at 42 °C for 60 min, and 70 °C for 5 min enzyme inactivation.

To quantify the expression of ER stress marker genes (s-XBP1/us-XBP1, ATF4, ATF6), the qPCR reaction was done using SYBR Green Real-Time PCR Master Mix (2×) (Ampliqon, Copenhagen, Denmark) on an ABI System (Applied Biosystems, Carlsbad, CA, USA). Primers are listed in Table 1. Reaction cycling conditions were 95 °C for 10 min followed by 40 cycles of 95 °C for 15 s, 60 °C for 30 s, and 72 °C for 30 s. In the present study, β-actin was employed as the housekeeping gene, and all genes were normalized to β-actin level using the 2^−ΔΔCt^ method.

### 4.9. Western Blot Analysis

Western immunoblotting was used for molecular assessment. First, a cold RIPA lysis buffer with Halt™ Protease and Phosphatase Inhibitor Cocktail (containing sodium fluoride, Sodium orthovanadate, β-Glycerophosphate, Sodium pyrophosphate, Aprotinin, Bestatin, E64, Leupeptin and EDTA) was added to the hippocampi and was homogenized by a Micro Smash homogenizer (MS-100). To collect protein-containing supernatant and to remove debris, the lysates were centrifuged at 13,000 rpm for 30 min at 4 °C. Protein concentrations were determined by the Bradford method. Equal amounts of proteins (50 µg) were then loaded and electrophoresed using 12% SDS-PAGE polyacrylamide gels and then transferred to PVDF membranes. After blocking with 5% BSA for 1 h at room temperature, the membranes were probed with primary antibodies overnight at 4 °C. The following day, the membranes were washed with TBS-T three times (ten minutes for each time) and then were incubated with horseradish peroxidase-conjugated antirabbit antibody for 1.5 h at room temperature. Immunoreactive bands were visualized by probing the blots with an ECL select kit. Finally, the radiographic films were scanned, and the protein band density was measured by Image J software (NIH, Bethesda, MD, USA). In some cases, blots were stripped before being re-probed with another primary antibody that had band interference (such as β-actin). In these cases, blots were incubated for 25 min at 50 °C in stripping buffer containing: 2% SDS-100 mM beta-mercapto-ethanol and 50 mM Tris, pH = 6.8.

### 4.10. Statistical Analysis

All the data are presented as mean ± standard error of the mean (S.E.M). The data were analyzed using the GraphPad Prism 6 Demo (GraphPad Software, San Diego, CA, USA) program. Data obtained from three days of training trials were analyzed by two-way repeated measures ANOVA. Results obtained from the probe and visible tests, as well as molecular results, were analyzed with one-way ANOVA. Tukey’s post hoc test was used for multiple comparisons. A difference between groups was considered significant when *p* < 0.05.

## 5. Conclusions

In conclusion, we showed that icv injection of TG triggered ER stress in the hippocampus of rats. The animals demonstrated activation of TNF-α, increased active caspase-3 and the ratio of Bax/Bcl2, P38, and brain IRS1307 phosphorylation after TG administration accompanied by impaired spatial learning and memory. These findings suggest the crucial role of ER stress in reducing cognition, which is mediated by neuroinflammation, apoptosis, impairment of MAPK, and brain insulin signaling. However, the detailed functional roles of those involved in ER stress-induced memory impairment mechanisms remain to be clarified.

## Figures and Tables

**Table 1:** Primer sequences (5′–3′) used for the quantitative polymerase chain reaction (qPCR). Spliced X-box binding protein 1 (s-XBP1), unspliced (us) XBP1, activating transcription factor-6 (ATF6), activating transcription factor-4 (ATF4).

Gene	Sequence	Amplicon (bp)	Reference
s-XBP1	F-CTGAGTCCGAATCAGGTCAG	59	[124]
	R-ATCCATGGGAGATGTTCTGG		
us-XBP1	F-GTCCGCAGCACTCAGACTAC	268	[124]
	R-ATGAGGTCCCCATGACAGA		
ATF6	F-CGAGGGGAGGTGTCTGTTTC	97	[125]
	R-GTCTTCACTGGTCCATGAGG		
ATF4	F-CTGAACAGCGAAGTGTTGGC	224	[124]
	R-TCTGTCCCGGAAAAGGCATC		

## Supplementary Materials

The following supporting information can be downloaded at: https://www.mdpi.com/article/10.3390/life12091374/s1, original western blot images.
