# Supplementary material for: Behavioral and Molecular Effects of Thapsigargin-Induced Brain ER- Stress: Encompassing Inflammation, MAPK, and Insulin Signaling Pathway
Source: Life (Basel). 2022 Sep 2;12(9):1374. doi: 10.3390/life12091374 (PMC9500646; doi:10.3390/life12091374)

Behavioral and molecular effects of Thapsigargin-induced brain ER- stress: Encompassing inflammation, MAPK, and insulin signaling pathway

Original blots presented in figures

Fig. 6.A: Bip

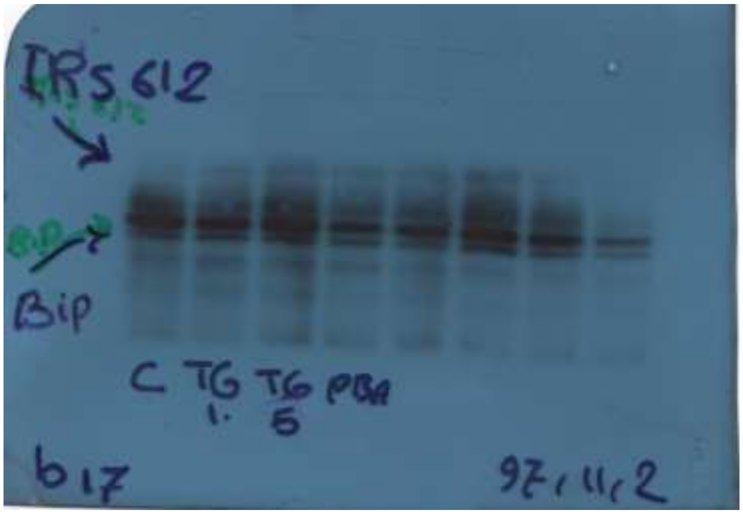

Fig. 6.A: Beta actin

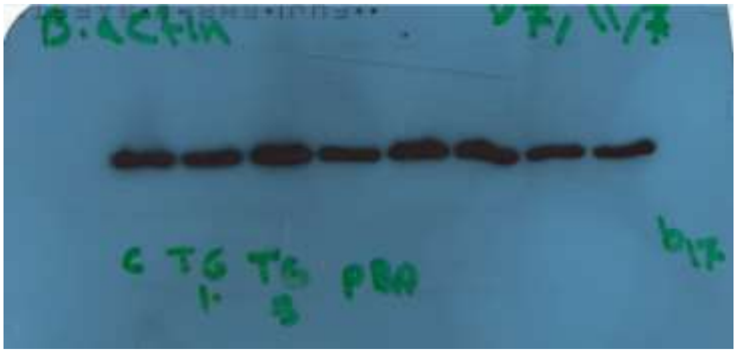

Fig. 6.B: CHOP

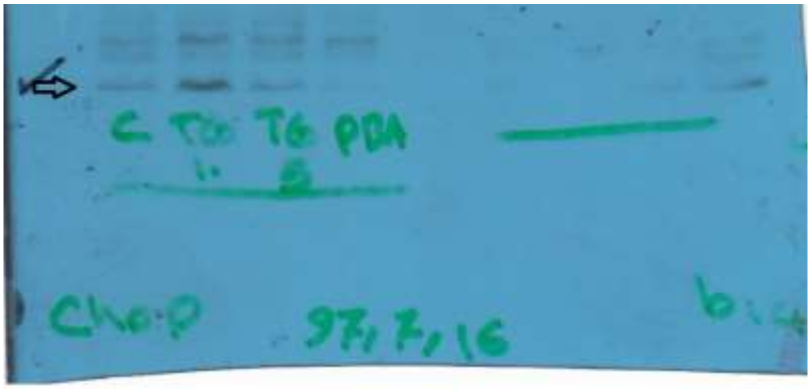

Fig. 6.A: Beta actin

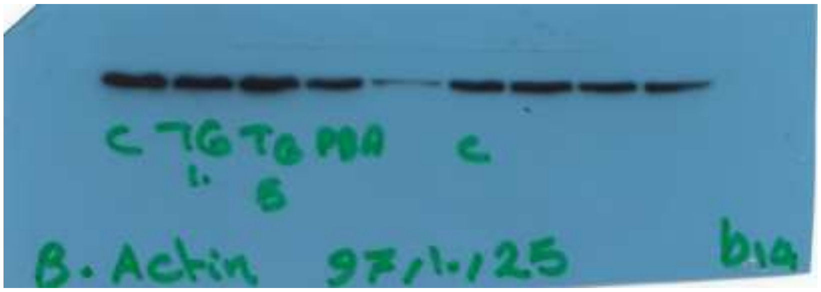

Fig. 7.A: Cleaved Caspase3 (gel2 blot4)

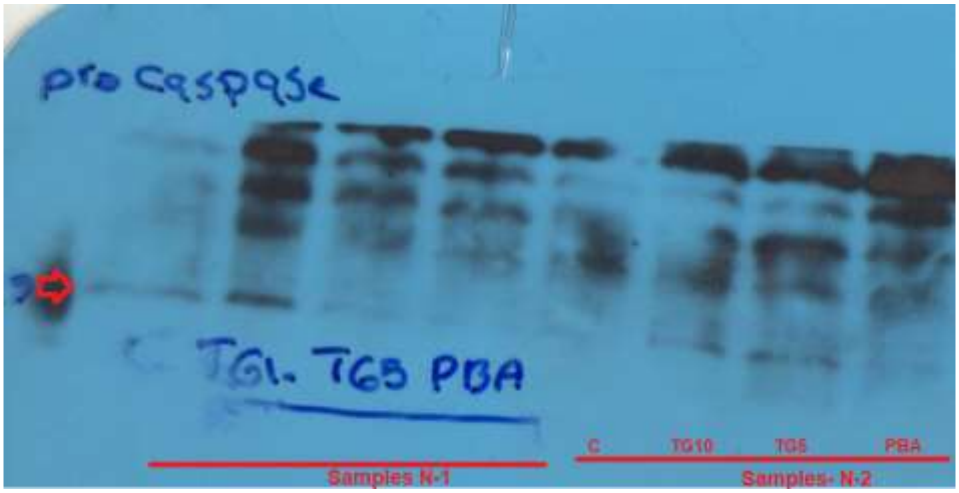

Fig. 7.A: Beta actin (gel2 blot3)

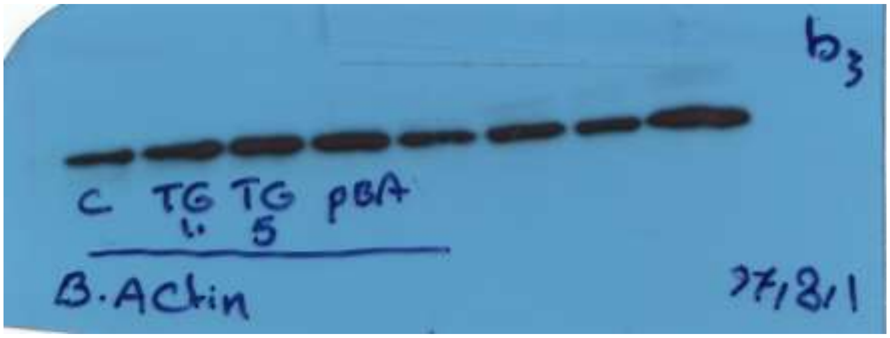

Regarding, the figure 7A, B and 9A, these blots are driven from the same blots (the same gel), therefore the same B Actin blot were presented for them. It is noteworthy to mention that, as it is evident in the original blots, two series of each samples were loaded on one gel (C-TG10-TG5-PBA--- C-TG10-TG5-PBA). After being transferred, according to the Prestained ladder, the blot form each gel was cut into two at cut point of 35Kd, one containing higher molecular weights (≥35Kd) and another lower molecular weights (≤35Kd) (for example blot 3, 4 from Gel2) were blocked and probed in parallel. That's the reason why the blot for B actin (MW;45) is named as blot-3 and Bax, Bcl2 and caspase named as Blot-4. These both are from the same gel2. The same is also true for TNF blot (17Kd). Blots 7, 8 are from Gel4.

Fig. 7.B: Bax (gel2 blot4)

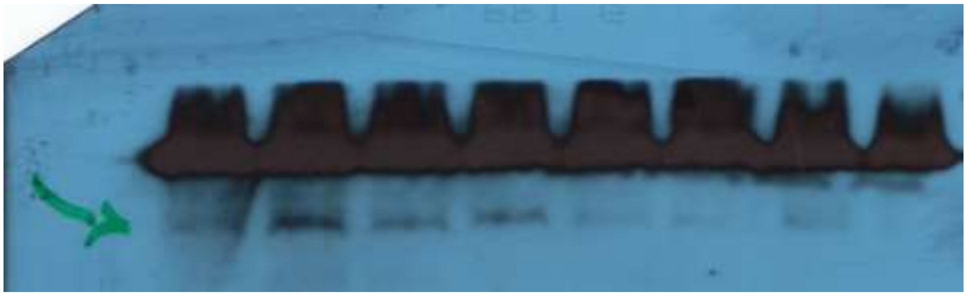

This blot was first probed with Bcl2 (26Kd) and then Bax (20Kd), without stripping, the Bax needed much more exposure time than Bcl2 to be revealed. This is why in Bax blot, Bcl2 is detected again which a very high density.

Fig. 7.B: Bcl2 (gel2 blot4)

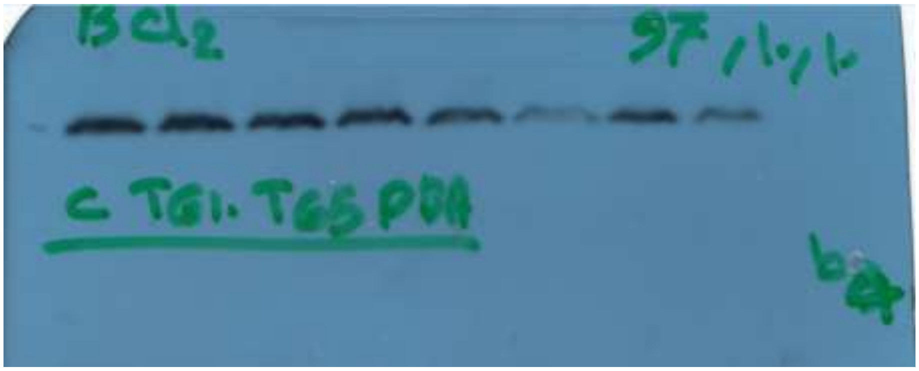

Fig. 8: TNF-α (gel4 blot8)

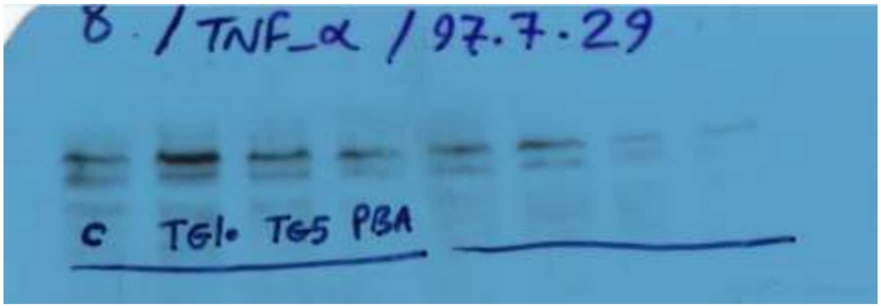

Fig. 8: Beta Actin (gel4 blot7)

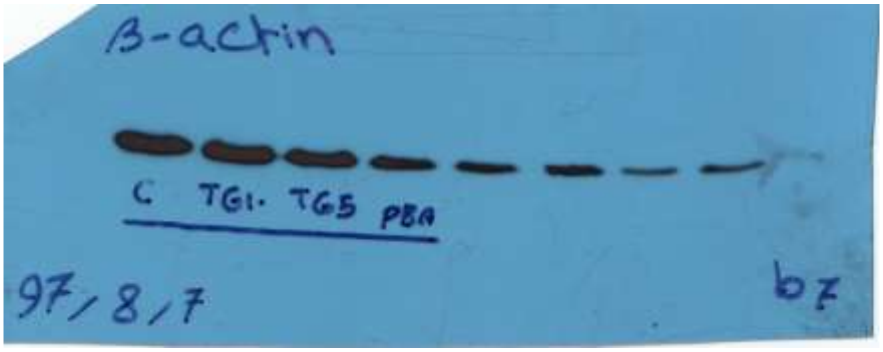

As explained earlier, blot 7 and blot 8 both were from the same Gel4, blot7 covered ≥35Kd bands and blot 8 covered ≤35Kd.

Fig. 9.A: p-JNK (gel2 blot3)

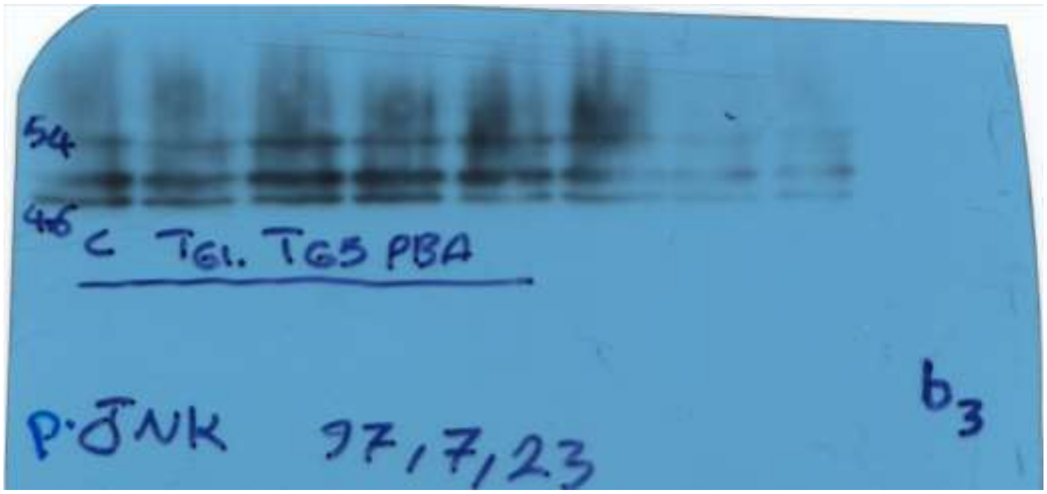

Fig. 9.A: T-JNK (gel2 blot3)

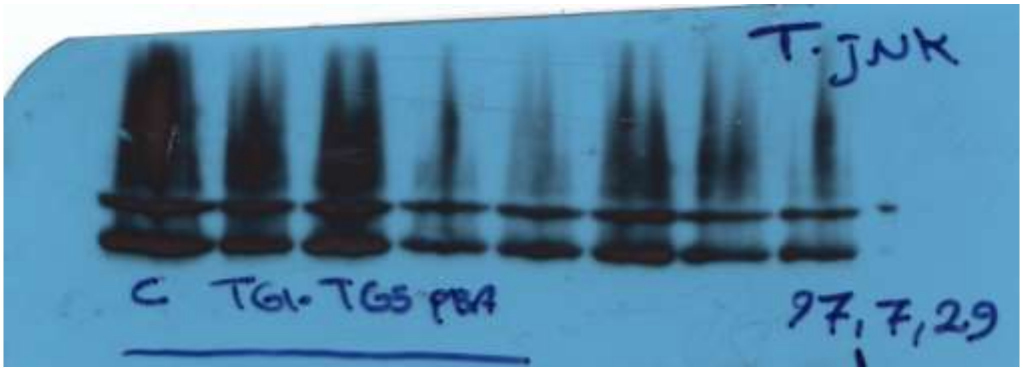

Fig. 9.A: Beta Actin

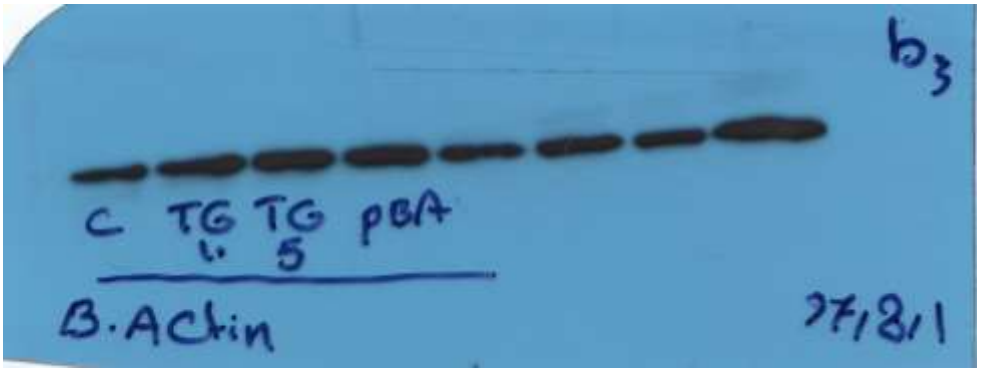

Fig. 9.B: p-P38 (gel1 blot2)

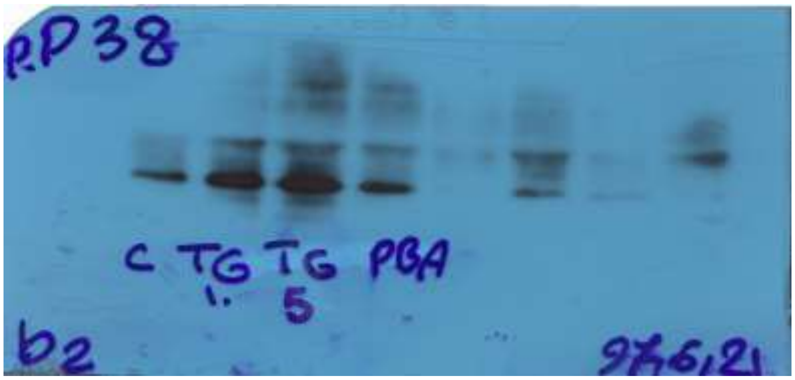

Fig. 9.B: T-P38

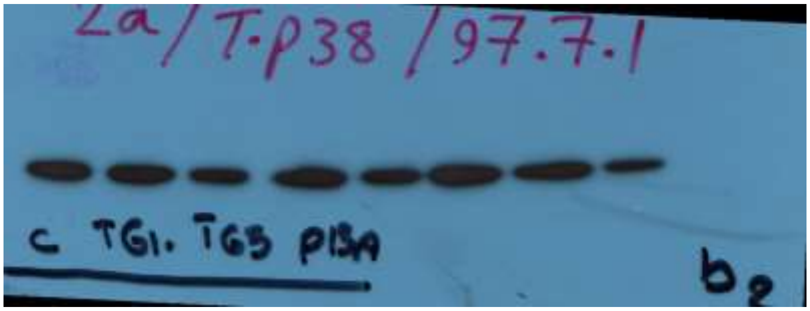

Fig. 9.B: Beta Actin

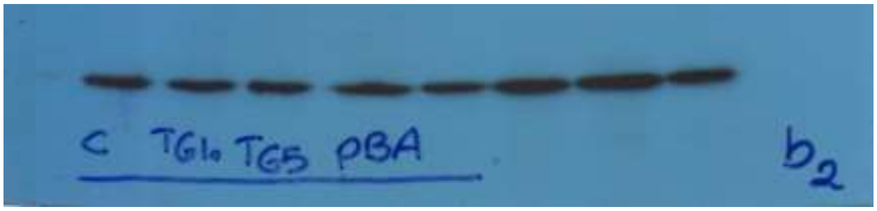

Fig. 10.A: p-IRS1

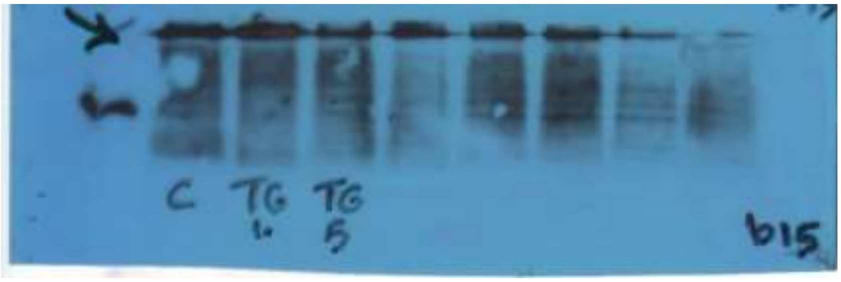

Fig. 10.A: T-IRS1

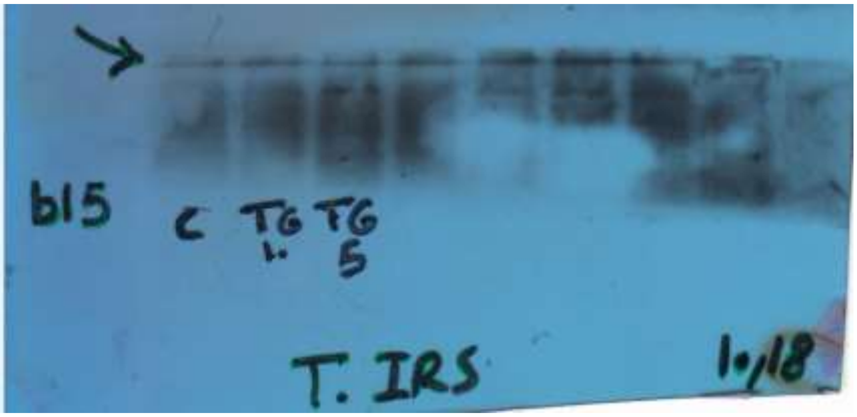

Other Original blots

Bip (N3,4)

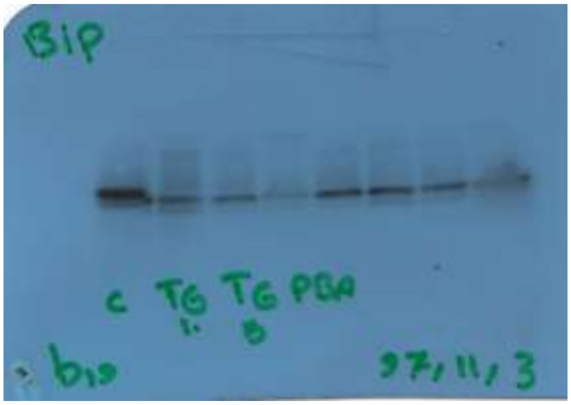

CHOP (N3,4)

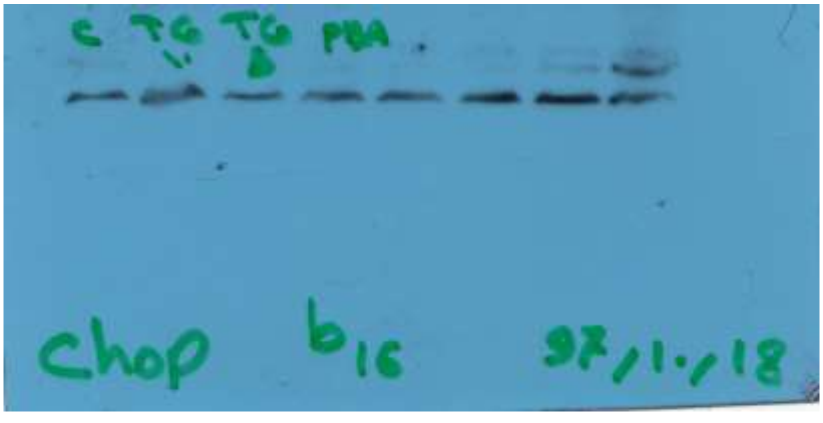

Cleaved Caspase3 (N3,4)

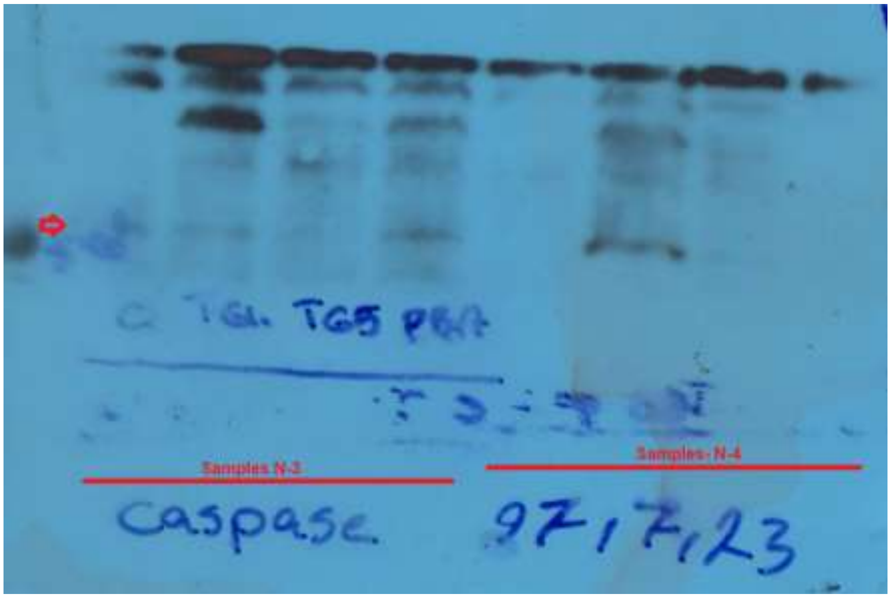

Bax(N3,4)

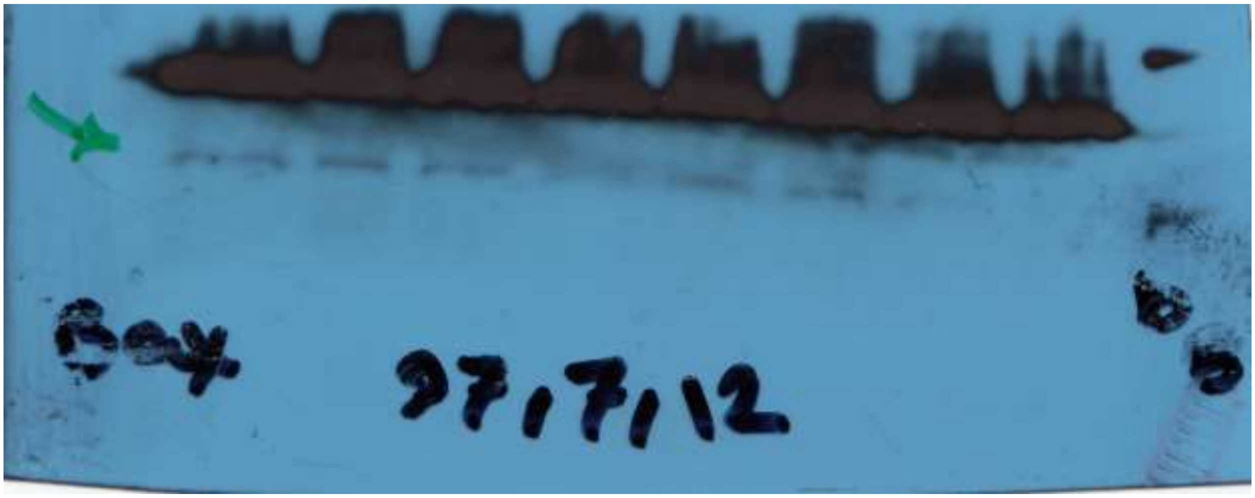

Bcl2(N3,4)

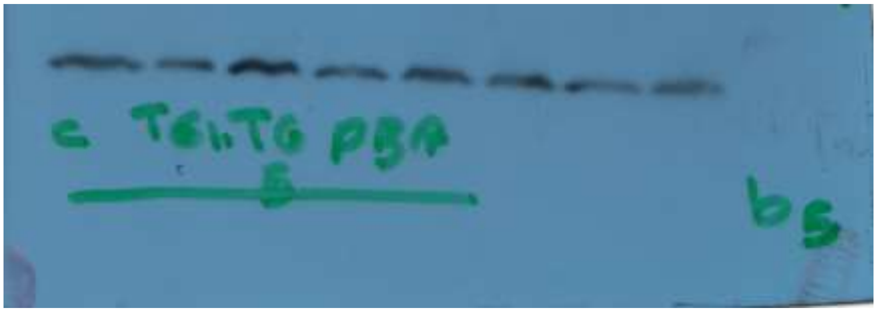

TNF-α(N3,4)

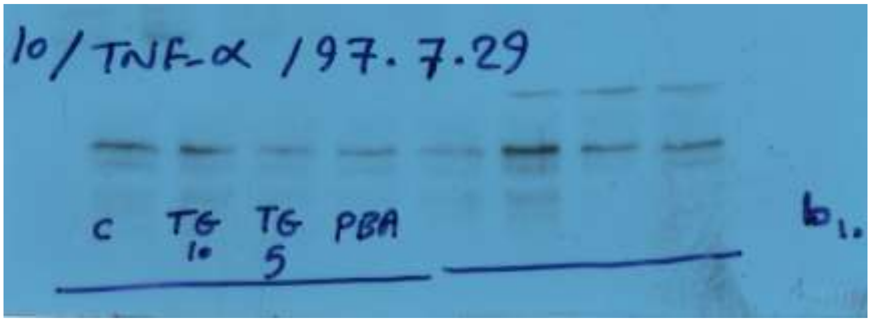

p-JNK (Blot3 N1,2 and blot6 N3,4)

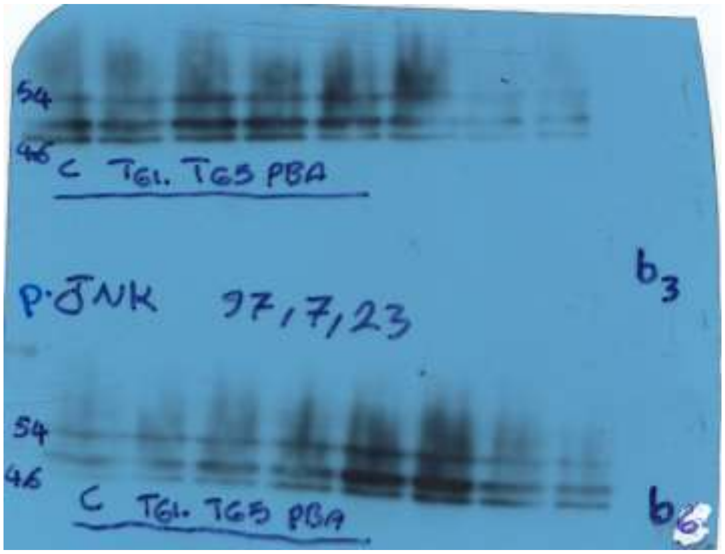

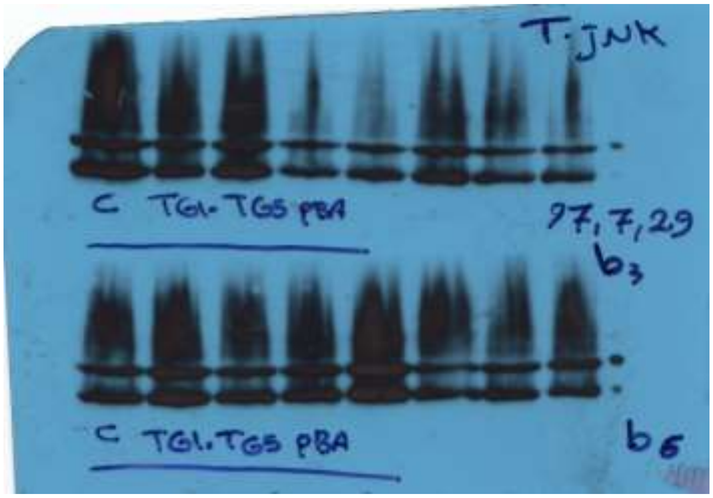

p-P38

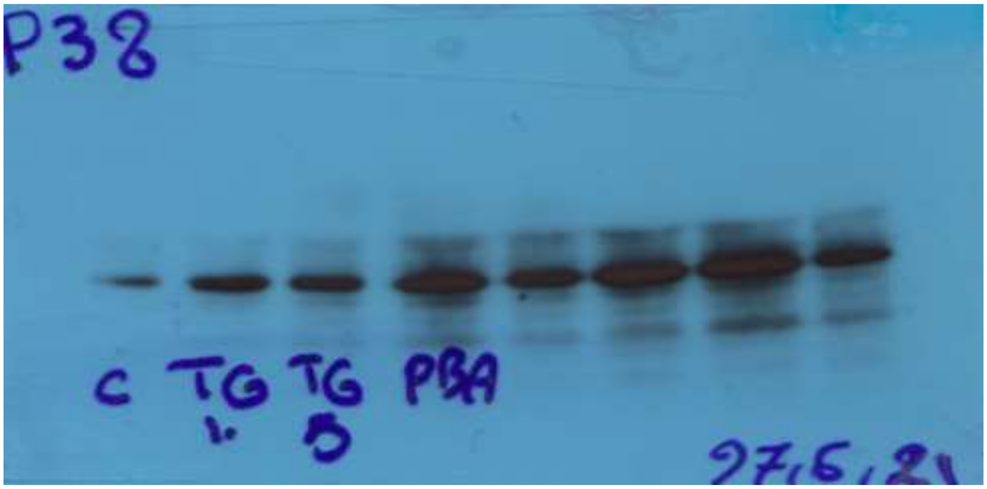

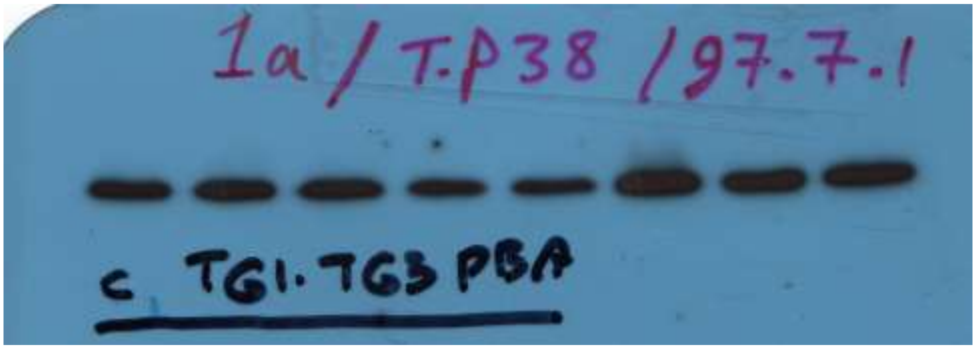

p-IRS1

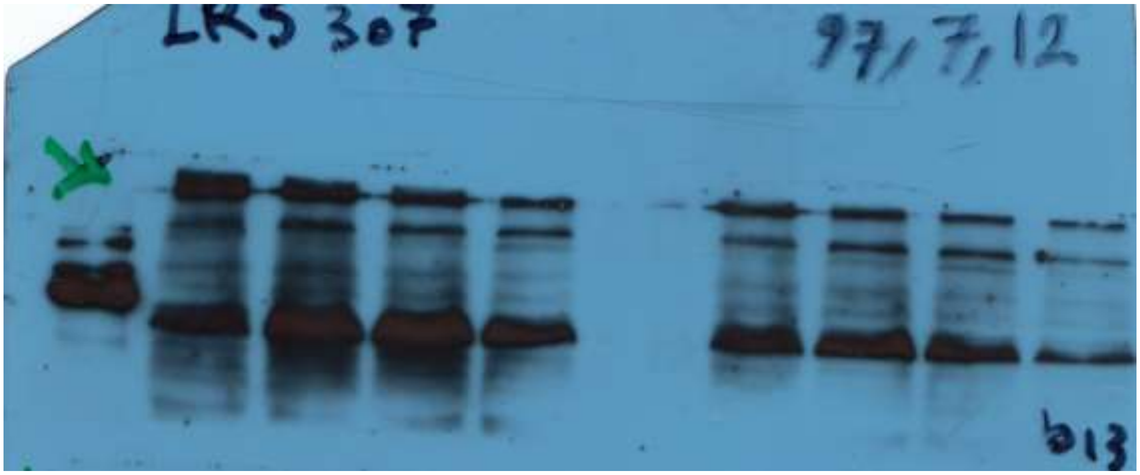

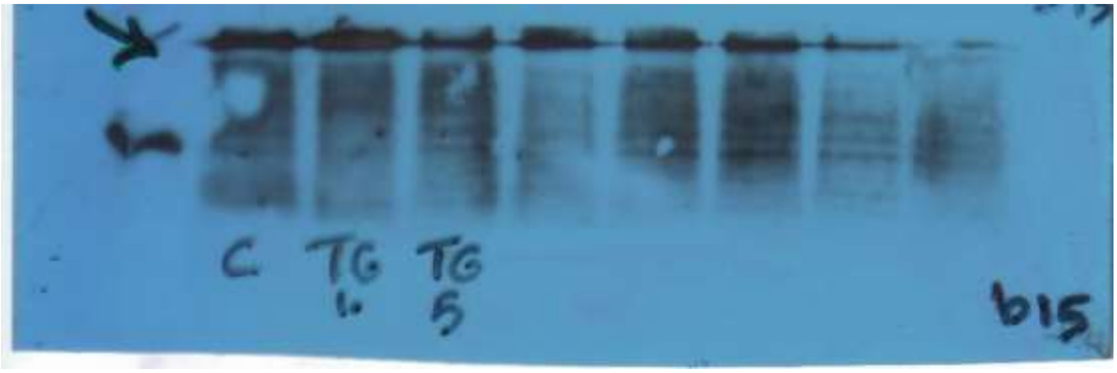

Supplement: Supplementary file 1 [file life-12-01374-s001.zip › life-1833299-supplementary.pdf]
